# Sperm chromatin accessibility’s involvement in the intergenerational effects of stress hormone receptor activation

**DOI:** 10.1038/s41398-023-02684-z

**Published:** 2023-12-08

**Authors:** Vincent Fischer, Miriam Kretschmer, Pierre-Luc Germain, Jasmine Kaur, Sergio Mompart-Barrenechea, Pawel Pelczar, David Schürmann, Primo Schär, Katharina Gapp

**Affiliations:** 1 Laboratory of Epigenetics and Neuroendocrinology, Institute for Neuroscience, Department of Health Sciences and Technology, ETH Zürich, Zürich, Switzerland; 2 Neuroscience Center Zurich, ETH Zürich and University of Zürich, Zürich, Switzerland; 3 Laboratory of Molecular and Behavioral Neuroscience, Institute for Neuroscience, Department of Health Science and Technology, Zürich, Switzerland; 4 Computational Neurogenomics, Institute for Neuroscience, Department of Health Science and Technology, Zürich, Switzerland; 5 Laboratory of Statistical Bioinformatics, University of Zürich, Zürich, Switzerland; 6 Center for Transgenic Models, University of Basel, Basel, Switzerland; 7 Department of Biomedicine, University of Basel, Basel, Switzerland

**Keywords:** Epigenetics in the nervous system, Epigenetics and behaviour, Physiology

## Abstract

Dexamethasone is a stress hormone receptor agonist used widely in clinics. We and others previously showed that paternal administration of dexamethasone in mice affects the phenotype of their offspring. The substrate of intergenerational transmission of environmentally induced effects often involves changes in sperm RNA, yet other epigenetic modifications in the germline can be affected and are also plausible candidates. First, we tested the involvement of altered sperm RNAs in the transmission of dexamethasone induced phenotypes across generations. We did this by injecting sperm RNA into naïve fertilized oocytes, before performing metabolic and behavioral phenotyping of the offspring. We observed phenotypic changes in discordance with those found in offspring generated by in vitro fertilization using sperm from dexamethasone exposed males. Second, we investigated the effect of dexamethasone on chromatin accessibility using ATAC sequencing and found significant changes at specific genomic features and gene regulatory loci. Employing q-RT-PCR, we show altered expression of a gene in the tissue of offspring affected by accessibility changes in sperm. Third, we establish a correlation between specific DNA modifications and stress hormone receptor activity as a likely contributing factor influencing sperm accessibility. Finally, we independently investigated this dependency by genetically reducing thymine-DNA glycosylase levels and observing concomitant changes at the level of chromatin accessibility and stress hormone receptor activity.

## Introduction

Diseases affecting mental health are often induced or aggravated by stress and persistently rank among the highest health burdens worldwide [1, 2]. The effects of stress hormones are mediated via their receptors, including the ubiquitously expressed glucocorticoid receptor (GR), among others. GR plays a pivotal role in regulating stress hormone levels via negative feedback in the hypothalamus pituitary adrenal gland axis [1]. At the molecular level, GR acts as a transcription factor (TF) that can homo- or heterodimerize upon activation by glucocorticoids and bind to the DNA to regulate the transcription of other genes, but also its own expression [3]. Aberrant activation of GR, such as that elicited upon chronic or traumatic stress, can have detrimental consequences on health and result in long-lasting neuropsychiatric diseases. Besides increasing neuropsychiatric disease risk in the directly exposed organism, stress and aberrant GR activation can under some circumstances also influence offspring phenotype, due to its potential to impact the molecular makeup of germ cells.

Over the past decade, studies using a variety of stressors have linked the transmission of behavioral or metabolic effects to alterations in sperm RNAs, including miRNA, lncRNA, tRNA fragments, ribosomal and circRNA [4–9]. While correlations of intergenerational impacts of stress with changes in sperm DNA methylation have also been reported [10, 11], causal evidence is restricted to sperm RNA and only one study could establish correlates to human conditions [12].

We previously showed that a single systemic administration of dexamethasone (Dex), an agonist of one of the main stress hormone receptors, GR, is sufficient to elicit a phenotype in the offspring [5].

This was accompanied by changes in sperm RNAs, suggesting that these altered sperm RNAs might be involved in the transmission mechanistically. While some of the affected RNAs were also found altered in other models and have been clearly described to affect embryonic gene expression via transposable elements, our model did not show this type of regulation. This opens the intriguing possibility that these changes might not be the main carrier of information across generations in our model.

Sperm chromatin transmits a few histones (1–10% in mouse) with their post-translational modifications, as a potential means to impact offspring gene regulation [13–16]. Retention of nucleosomes in sperm is correlated with the establishment of DNA methylation-free regions in the early embryo [17]. *Gcn5* mediated histone acetylation was proven to be necessary for histone eviction and sperm fertility [18]. A specific set of histone post-translational modifications (hPTMs) in sperm have been shown necessary for normal offspring development [19] and their absence has been proven to aggravate diet induced effects on the health of the progeny [20]. In line with this, several studies showed that some hPTMs are sensitive to other environmental exposures such as drugs, hepatic injury and diet [21–24] and undergo extensive remodeling during epididymal transit [25]. In addition, recent work suggested that chromatin accessibility can be perturbed by environmental exposures [26–28]. Alteration of sperm nucleosome retention at various loci [through dysregulation of poly(ADP-ribose) metabolism during spermiogenesis] is associated with differential expression of a subset of these genes encoded in these regions in the two-cell embryo [29]. Hence, environmentally triggered perturbation of chromatin accessibility might give rise to new loci that retain histones and hPTMs in the otherwise compacted sperm chromatin and could contribute to the intergenerational effects of stress exposure.

Here we aimed to find the mechanistic underpinnings of the effects of paternal pharmacological stress hormone receptor activation on the offspring. We first interrogated the functional contribution of changes in sperm RNA following paternal Dex administration and found that Dex induced changes in RNA are functional but not fully deterministic regarding the transmission of Dex effects. We then tested chromatin accessibility as an alternative information carrier across generations to conclude that at least some intergenerational effects are likely related to sperm chromatin accessibility, and that this is likely attributed to stress hormone receptor activity at the affected loci.

## Results

### The role of sperm RNA in mediating effects of dexamethasone

**Fig. 1:**
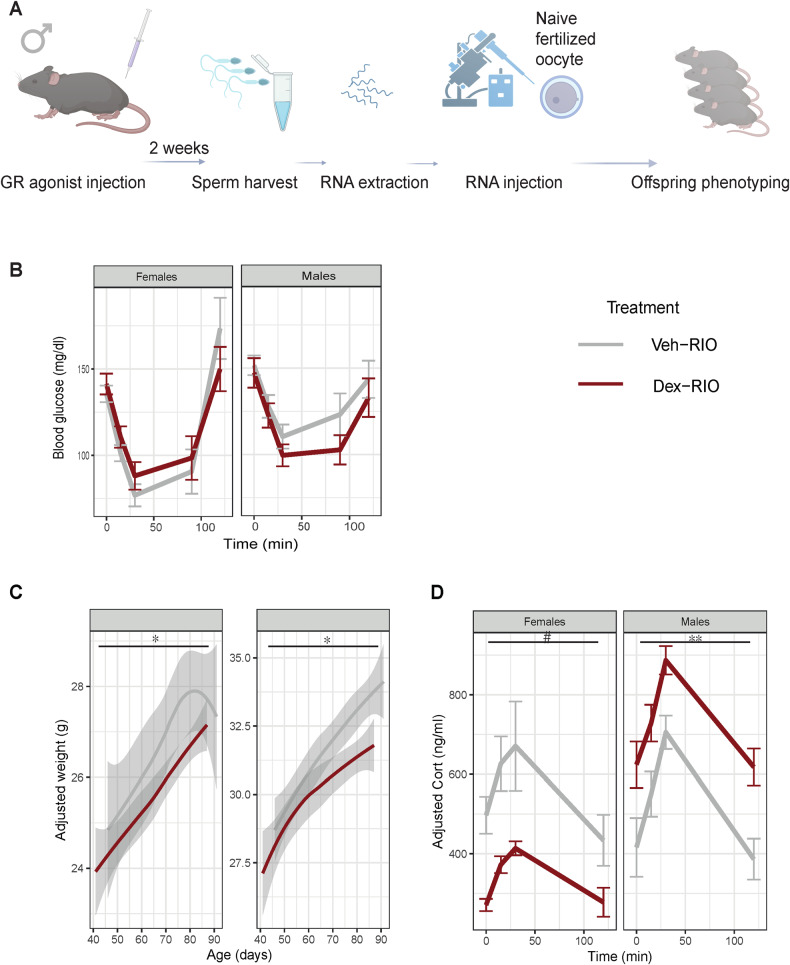
The role of sperm RNA in mediating effects of Dexamethasone injection across generations. **A** Scheme depicting experimental design of GR-agonist (Dex) treatment, sperm collection, RNA extraction, RNA injections, offspring generation and offspring phenotyping. Adult males received either a Veh or Dex injection followed by 2 weeks of rest to allow sperm to pass through the epididymal tract. Sperm was then harvested for RNA extraction and subsequent injection into naïve fertilized oocytes. Embryos were implanted into foster mothers to generate offspring that were phenotyped in adulthood. **B** Insulin tolerance test in offspring resulting from RNA injections into fertilized naïve oocytes with sperm from GR-agonist treated males and vehicle treated males showed no significant group difference in the effect of insulin treatment on glucose response using repeated measurement ANOVA analysis of the insulin tolerance test, with no significant covariates (F(1, 59) = 0.022, *p* = 0.883). Consistent with the literature [91, 92], we detected a generally significant effect of sex on insulin tolerance, with females being more insulin sensitive than males (F(1, 59) = 9.793, *p* = 0.003). **C** Line graph depicting body weight of Dex-RIO over time using litter size as a covariate, reveals a significant interaction between time sex and treatment (F(2.533, 76.003) = 3.279, *p* = 0.032) and an interaction between time, sex and treatment (F(2.533, 76.004) = 3.635, *p* = 0.022) for body weight. Additionally, there is a significant sex effect (F(1, 30) = 35.800, *p* = 0.000) and a significant treatment effect (F(1, 30) = 4.475, *p* = 0.043). **D** Corticosterone levels in blood sampled by tail prick at baseline, in response to physical restraint and during the recovery phase in RIO using date of weaning and litter size as covariates revealed a significant group effect over time (F(1, 6) = 17.530, *p* = 0.006) in males and a nearly significant treatment effect in the opposite direction (F(1, 8) = 4.357, *p* = 0.070). Error bars (gray shaded area) represent standard error of the mean, *p < 0.05 interaction. **p < 0.01 group effect, #*p* < 0.1 group effect.

We previously detected a range of alterations in the abundance of small (tRNA fragments, miRNAs and ribosomal RNA fragments) and long RNAs (coding, non-coding and circRNAs) in response to an acute Dex exposure in mature sperm [5]. Whilst we inferred a link between sperm circRNAs, offspring embryonic gene expressions, and the transmission of some altered tRNA fragments, causal data were missing. We therefore extracted sperm RNA from Dex and vehicle (Veh) treated males at the same time when sperm was previously harvested for molecular analysis and in vitro fertilization (IVF) and generated a cohort of animals using sperm RNA injection into fertilized naïve oocytes. We chose the total RNA fraction for RNA injections into naïve fertilized oocytes (Fig. 1A), since previous studies have highlighted complementary functions of different classes of sperm RNA in the transmission of environmentally induced phenotypes [9, 30, 31]. The injected oocytes were reimplanted into pseudopregnant females to generate live offspring for phenotyping in adulthood.

Previous phenotyping of IVF offspring generated with sperm of Dex and Veh treated sperm had revealed significant effects on insulin sensitivity, body mass index and glucose tolerance [5]. Thus, we assessed the same characteristics in the RNA injected offspring (RIO). Interestingly, we observed no alterations in insulin sensitivity in male and female RIO (Fig. 1B). These findings raised the hypothesis that alterations in sperm RNA abundance are not the main carrier of insulin signaling related phenotypes in our model across generations. Absence of the insulin tolerance phenotype might be related to insufficient amounts of sperm RNA - hence we additionally assessed the body weight of the resulting animals and tested them for glucose tolerance, corticosterone levels in response to restraint, and risk-taking behaviors. We observed a significant sex dependent decrease in weight in the RIO (Fig. 1C). While this is in line with a change in BMI observed in IVF offspring, the change is in the opposite direction. This suggests that sperm RNA from Dex injected males can induce an altered trajectory of offspring phenotype, but that this trajectory is not identical to the one elicited as a whole by germ cells of Dex injected fathers (Supplementary Fig. 1A). Assessment of blood glucose in response to glucose injection also showed a consistent time and sex dependent change in glucose tolerance (Supplementary Fig. 1B), again not mimicking the direction of change in the IVF offspring [5]. This emphasizes the functionality of Dex altered sperm RNA in impacting the metabolic phenotype of the offspring, yet clearly indicates a differing trajectory in offspring sired by Dex injected fathers.

To further investigate the altered sperm RNA induced trajectory, we subjected animals to a restraint stress and assessed their corticosterone levels both during and following the acute stressor. We found a decrease in hormone levels in females and an increase in males (Fig. 1D, Supplementary Fig. 1C), indicating robust consequences of altered sperm RNA on stress responsiveness. Such alterations in stress responsiveness might indicate behavioral changes in stressful situations. We therefore further explored anxiety levels and indeed observed a sex independent reduction in the time RIO spent in the center of the field during an open field test (Supplementary Fig. 2A), indicating an anxiogenic effect. The increased anxiety was accompanied by a sex independent decrease in the distance covered (Supplementary Fig. 2B), potentially hinting towards overall reduced activity. The latter idea was corroborated by a significant overall reduction in the distance moved during a light dark box test, independent of sex (Supplementary Fig. 2D), in the absence of any effect on the time animals spent in the dark compartment of the box (Supplementary Fig. 2C).

Collectively, the phenotyping clearly indicates regulatory functionality of the affected levels of sperm RNA. The discordance of the exact phenotype observed in IVF offspring and RIO strongly suggests that either this is because sperm RNA injection experiments do not capture the effect of downregulated sperm RNA or because additional sperm epimodifications are at play.

### The effects of dexamethasone on alternative information carriers in sperm

Glucocorticoid hormones have been shown to directly impact chromatin remodeling during rat development and in vitro hepatoma cells [32]. At that time, a pathway for active demethylation was not fully elucidated yet. A direct relationship between GR and remodeling via active demethylation was proposed by a prominent study that demonstrated the involvement of DNA double strand breaks, hence suggesting a role of DNA repair mechanisms in this remodeling [33]. Therefore, we decided to examine whether glucocorticoid signaling might directly affect chromatin accessibility in sperm. To test this, we generated ATAC-seq data from sperm of age matched male control mice and males that had received an injection of the glucocorticoid agonist Dex two weeks earlier and compared the respective chromatin accessibility. This time window corresponds to the timing of sperm collection when generating offspring using in vitro fertilization, and when the small and long (non-)coding RNA had been profiled, allowing for epididymal transit of exposed germ cells. We then compared chromatin accessibility between the two groups. The ATAC-seq data showed the expected distribution of nucleosome-free, mononucleosome and polynucleosome sized regions (Supplementary Fig. 3A).

**Fig. 2:**
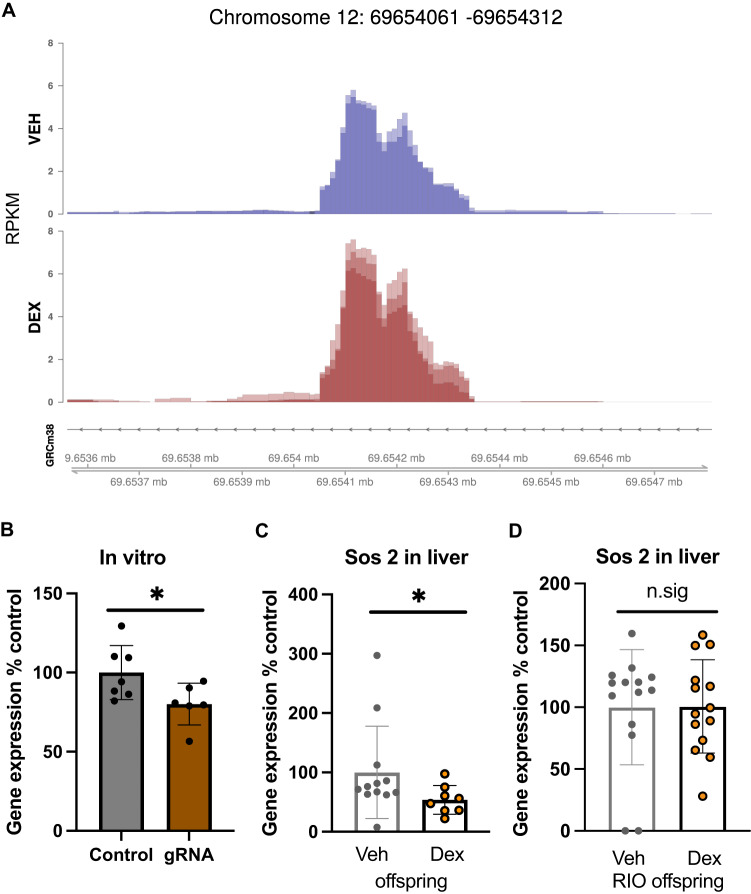
Effects of GR-agonist treatment on chromatin accessibility at the single gene level. **A** *Sos2* tracks depicting accessibility signals in sperm of GR-agonist treated males (*n* = 3) and vehicle treated males (*n* = 2) reveal decreased accessibility in the samples of agonist treated males **B** Gene expression of *Sos2* in N2a cells in vitro following dCas-KRAB treatment directed towards the affected locus indicates a downregulation of *Sos2* mRNA upon dCas-KRAB targeting against the locus (control *n* = 7, KRAB-dCas *n* = 6, t(11) = 2.32, *p* = 0.04). **C** Gene expression of *Sos2* in liver of offspring resulting from IVF with sperm from GR-agonist treated males and vehicle treated males shows a decrease in *Sos2* mRNA in the offspring of agonist treated animals (Veh offspring *n* = 12, Dex offspring = 8, *U* = 21, *p* = 0.04). **D** mRNA levels of *Sos2* in liver of offspring resulting from RNA injections into fertilized naïve oocytes with sperm from GR-agonist treated males and vehicle treated males as assessed by q-PCR did not show any differences between groups (Veh-RIO offspring *n* = 14, Dex-RIO offspring *n* = 14, t(26) = 0.04, *p* = 0.97). Error bars represent standard error of the mean, *p < 0.05.

Inspection of differential accessibility at a single-region-level showed several loci with putative changes, of which only two overlapped with protein coding genes, one of which was *Sos2* (Fig. 2A). Interestingly, *Sos2* can regulate insulin signaling [34], a process we knew was affected in IVF offspring of Dex exposed fathers [5]. We therefore hypothesized that the observed alteration might be implicated in affecting *Sos2* expression in the progeny of Dex injected fathers. To first test whether the affected region could at all be relevant in regulating expression of the affected gene, we tethered the epigenetic modifier KRAB to the locus using the deactivated Cas9 (dCAS9) system in vitro to induce silencing marks. Q-RT-PCR of Neuroblastoma N2a cells transfected with KRAB-dCas9 and a guide directed against the locus confirmed a modest but significant decrease in the expression of the *Sos2* gene in experimental samples (Fig. 2B), suggesting regulatory relevance of the targeted region. Hence, we assayed *Sos2* expression in the IVF offspring of Dex injected fathers. Intriguingly, Q-RT-PCR analysis of *Sos2* revealed a significant downregulation of the gene in the liver (Fig. 2C), the prime site of *Sos2* expression, and a strong trend for a downregulation in the kidney (Supplementary Fig. 3B).

If the insulin phenotype resulted from changes in chromatin accessibility and not the alterations of sperm RNA, *Sos2* expression should be unaltered in the liver of the offspring resulting from sperm RNA injections of Dex treated males into fertilized naïve oocytes (Dex-RIO). Consistent with this assumption, we did not observe changes in *Sos2* expression in the liver of these animals (Fig. 2D). In summary, RNA injections could not replicate the Dex induced gene regulation of *Sos2* in the Dex-RIO animals. This implies that GR activation mediated modulation of chromatin accessibility in sperm is a potential means of conveying information to the offspring.

### The effect of dexamethasone on chromatin accessibility globally, and at specific genomic features

**Fig. 3:**
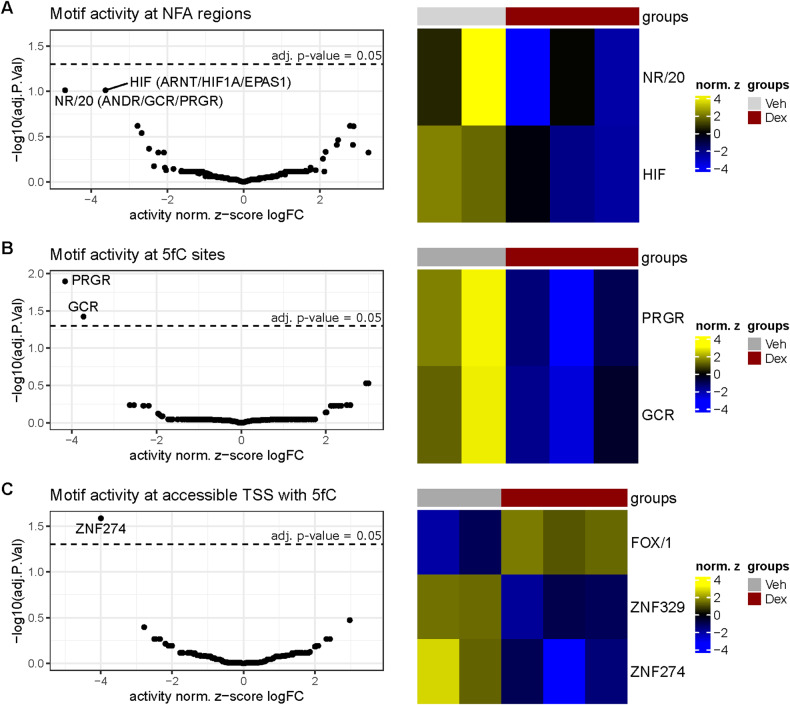
A potential link between open chromatin and GR. **A** Volcano plot and heatmap of differential motif archetype activity in sperm of GR-agonist treated males versus vehicle treated males assessed by ATAC sequencing depicts two archetypes with FDR corrected strong trend for differential accessibility. **B** Volcano plot and heatmap of differential motif activity in sperm of GR-agonist treated males versus vehicle treated males restricted to 5fC sites shows FDR corrected significant altered accessibility of PRGR and GCR = GR motifs at 5fC sites. **C** Volcano plot and heatmap of motif archetype activity in sperm of GR-agonist treated males versus vehicle treated males restricting to accessible TSS with 5fC reveals FDR corrected significant differential accessibility at the ZNF274 motif. Significance level is shown by horizontal line depicting the *p*-value of 0.05 following FDR correction.

We were further interested in whether GR activation would impact accessibility at a broad genome-wide scale. Overall, we did not observe widespread changes in nucleosome-free accessibility across the genome (Supplementary Fig. 4A, B). We therefore asked whether accessibility changes might coincide with certain genomic regions with specific features, instead of being equally affected across the genome. Open chromatin regions allow for the binding of TFs, and TF binding can be inferred from the presence of TF binding motifs in a given genomic region. Thus, we inspected chromatin accessibility changes specifically at TF binding sites including the motif family containing the glucocorticoid receptor response element (GRE) motif that binds GR. The GR homo- or heterodimerizes when activated by corticosteroid, or synthetic Dex binding, and is known to possess some pioneering TF characteristics, in that it can modify chromatin states [3]. Indeed, the accessibility of several motif archetypes showed a strong trend for alterations starting with the NR/20 motif family which includes the GRE motif (Fig. 3A), indicating decreased accessibility specifically at these sites following Dex treatment (Fig. 3A). Importantly, comparison of accessibility between the sperm of Dex and Veh injected males at sites marked by a DNA modification produced by 5-Hydroxymethylcytosine (5hmC) via the ten-eleven translocation enzymes (TET), namely, 5-Formylcytosine (5fC), pinpoints the progesterone receptor (PRGR) response element (PRE) and GRE motif with statistically significant changes for altered activity (Fig. 3B). Honing in on the functionally relevant transcriptional start sites (TSS) with 5fC, we could distinguish two clusters with and without accessible TSS (Supplementary Fig. 4C). Comparing motif accessibility at accessible TSS containing 5fC at the motif archetype level (grouping similar motifs together) between Dex and Veh, revealed ZNF274 as the one significantly affected archetypes of TF binding sites (Fig. 3C). This raised the question: does the specific capacity of regions marked with 5fC change their ability to respond to chromatin remodeling?

### Association between 5fC and histone retention at functionally relevant loci

**Fig. 4:**
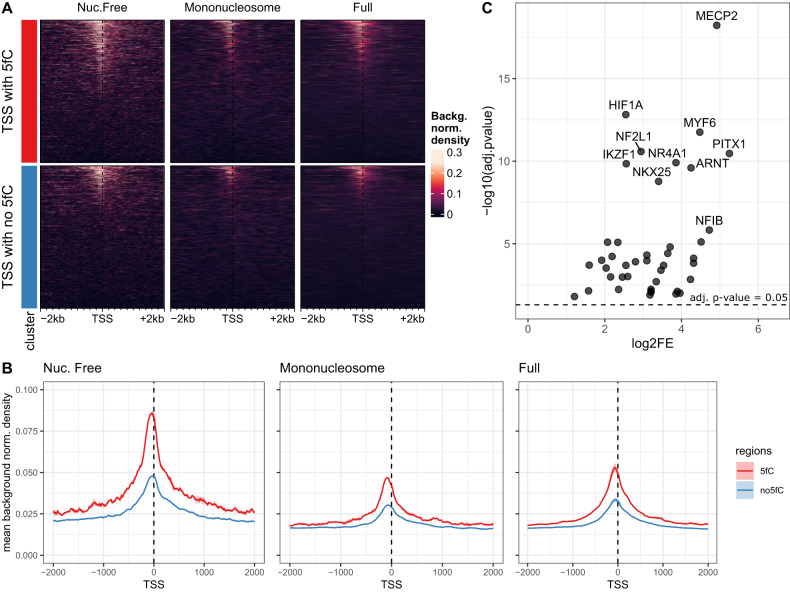
Interplay of 5fC and chromatin accessibility in sperm. **A**, **B** Chromatin accessibility assessed by ATAC sequencing from (**A**) mouse sperm data at TSS with or without 5fC (±2kb) (*n* = 2) shows increased accessibility at a range of TSS with 5fC versus a range of TSS without 5fC. Sites without 5fC (no 5fC) were randomly sampled to match the 5fC site number. **B** Increase of accessibility is further confirmed by the mean accessibility of all 5fC sites compared to all no5fC sites (**C**) Motif enrichment analysis of 5fC sites proximal to TSS versus methylated promoter regions in sperm (−1000bp,+100 bp) suggests an enrichment of several motifs of proteins related to DNA methylation. Due to the low number of background regions, the fold change couldn’t be determined for all given motifs, which then were excluded. Significance level is shown by horizontal line depicting the *p*-value of 0.05 following FDR correction. The top 10 most significant motifs are labeled with their respective names.

Gao et al. suggested a link between 5fC and chromatin accessibility around TSS when analyzing DNAse-seq data of inner cell mass [35]. Encouraged by this observation in humans, we generated ATAC sequencing data from mouse sperm (Supplementary Fig. 3A) and used the mouse single cell 5fC data from Zhu et al. [36] to find that this pattern of increased accessibility at TSS with 5fC versus TSS without 5fC (no5fC) was detectable in mouse sperm (Fig. 4A, B). Motif enrichment analysis of TSS with 5fC versus methylated promoters showed several DNA methylation (DNAme) readers and other DNA modification remodeling related TFs (Fig. 4C). To further explore putative TF binding in open chromatin in sperm, we compared sites with 5fC proximal to TSS (Supplementary Fig. 5A), but also overall 5fC sites (Supplementary Fig. 5B, C) to sites without 5fC proximal to TSS and overall sites without 5fC respectively. Both comparisons confirmed an enrichment for motifs that can bind TFs that function as DNAme readers such as MeCP2 and MBD2, potentially indicating directed remodeling of the DNA methylation status and its derivatives at those sites.

### Interplay of Thymine-DNA glycosylase, glucocorticoid receptor activity and sperm chromatin accessibility

**Fig. 5:**
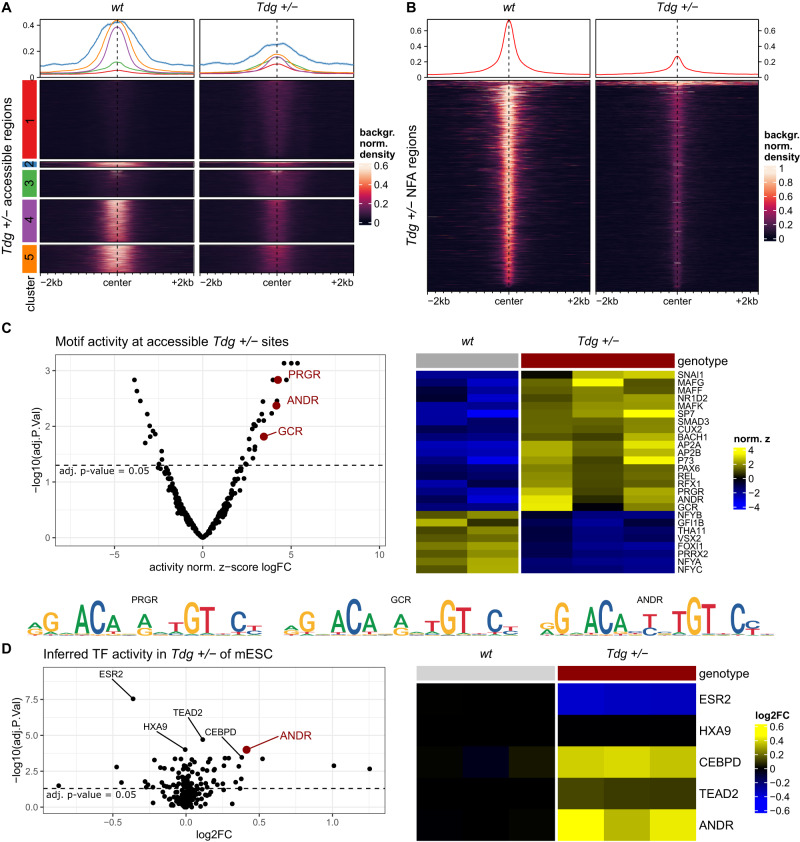
Effects of genetic manipulation of *Tdg* on sperm chromatin accessibility. Heatmap of ATAC sequencing data of 2 wildtype control and 3 *Tdg* ± sperm samples across (**A**) accessible regions based on the 3 *Tdg* ± samples (**B**) nucleosome-free accessible regions based on 3 *Tdg* ± samples reveals widespread decreased accessibility in *Tdg* ± samples, yet further indicates 2 clusters (clusters 1, 3) of regions with increase in accessibility **C** heatmap and volcano plot of differential motif activity (in nucleosome-free accessible regions) of *Tdg* ± versus control sperm (**C**) indicates a range of differential accessible motifs, including PRGR, ANDR and GCR = GR. **D** Heatmap and volcano plot of inferred TF activity of *Tdg* ± versus control ESCs reveals reduced ANDR activity in *Tdg* ± samples (top 5 significant TFs are shown with their respective names). Significance level is shown by horizontal line depicting the *p*-value of 0.05 following FDR correction. Nuc. Free nucleosome-free, NFA regions nucleosome-free accessible regions, ESCs mouse embryonic stem cells.

5fC can undergo base-excision repair by thymine-DNA glycosylase (TDG). Interestingly, it was also previously shown that TDG can bind GR [37]. Therefore, we aimed to alter TDG levels to investigate the potentially converging consequences of altered TDG levels and GR activation on sperm chromatin. It was shown that genetic depletion of TDG shows heterogeneous increases of 5fC in E11.5 embryos [38] and embryonic stem cells [39]. In comparison to most somatic cells, 5fC levels are low in mature sperm [40, 41]. Homozygous *Tdg* deficient animals show embryonic lethality [42–44], therefore we used heterozygous knockout animals (*Tdg* ±) to assess its effects on chromatin accessibility. We performed ATAC sequencing with three replicates of heterozygous *Tdg* ± sperm and two replicates of age matched *Tdg* ± wildtype (wt) controls. We first corroborated the correct size distribution, including nucleosome-free-, mononuclesome-, and multinucleosomal-sized fragments, of ATAC-seq reads (Supplementary Fig. 6a). We observed an overall decrease in accessibility in *Tdg* ± samples, across accessible sites (reflecting sites of histone and/or histone and TF retention) identified in control sperm (Supplementary Fig 6b). Focusing on regions found to be accessible in the *Tdg* ± samples, we observed that the signal was not uniformly decreasing, and identified both clusters with increased (clusters 1, 3) and decreased (clusters 2, 4, 5) accessibility (Fig. 5A). Since the increased accessibility is only apparent when focusing on regions found to be accessible in the *Tdg* ± samples and not when focusing on consensus accessibility across all samples (Fig. 5B), we concluded that clusters 1 and 3 represent sites that were indeed inaccessible in wt sperm, and thus are not captured by the ATAC sequencing in control samples. Such an increase might indicate that lower TDG levels cause an increase in 5fC, and that 5fC directly plays a role in determining chromatin accessibility, although we cannot exclude the additional influence of 5-Carboxylcytosine (5caC). If one expects a correlation between the presence of 5fC and accessibility, then changes should occur in those sites that in control conditions lack 5fC, no change is however expected at 5fC sites. Indeed, analysis of known 5fC sites in sperm showed little or no overall accessibility change (Supplementary Fig. 6C).

Since we also observed clusters with decreased accessibility (Fig. 5A) we speculated that haploid sperm TDG might rather play a role in TF recruitment to orchestrate 3D-reorganization as has been shown in the context of cancer cell lines in response to estradiol [45]. An additional such independent mechanism might be indeed related to the TDG mediated oxidation of 5caC conversion to cytosine, since chromatin structure associated alterations in CTCF binding were shown to be 5caC dependent [46]. Honing back in on TSS with 5fC also further corroborated a decrease in accessibility (Supplementary Fig. 6D), arguing against a linear relationship between levels of TDG, 5fC and accessibility.

To further investigate a potential contribution of TDG independent of 5fC, we subjected the accessibility sites in the *Tdg* ± samples to a motif activity analysis at the nucleosome-free level. Comparison of accessible sites of *Tdg* ± between the mutant and wt controls revealed a range of significant TF motifs (Fig. 5C). One of the highly affected motifs is the GRE motif activity (Fig. 5C).

To gather further evidence on the potential interplay of TDG and GR signaling we turned towards mouse embryonic stem cells (ESCs) and assessed gene expression in *Tdg* ± ESCs versus wt ESCs. Using next generation sequencing following ribosomal depletion, we found significant differences in gene expression between the two groups of samples (Supplementary Fig. 7A,B). Further inference of TF activity elucidated significant effects on the activity of several motifs, with strongest alterations in the androgen receptor (Fig. 5D). Interestingly, the androgen receptor (ANDR) and the GR bind to very similar motifs and their recruitment depends mostly on different cofactors [47]. Since our and other ESCs gene expression data showed that ESCs are void of ANDR transcripts, this could indicate that GR, that is indeed expressed in ESCs, is activating genes that are in other cell types regulated by the androgen receptor. Hence these data at least support the concept that altered levels of TDG affect steroid receptor mediated gene expression regulation via chromatin accessibility in ESCs. These results suggest a relationship between TDG, steroid receptors and chromatin accessibility in general and further substantiate a mechanistic contribution to the intergenerational effects of Dex exposure.

## Discussion

Here we show that altered sperm RNA payload does not necessarily equal the transmission of a paternal stress exposure elicited intergenerational phenotype. Several rodent studies, including our own previously demonstrated that changes in sperm RNA payload following different kinds of chronic stressors are sufficient to mimic an intergenerationally transmitted phenotype when injected into naïve fertilized oocytes [4, 6, 9, 30, 31, 48]. As a consequence, sperm RNA was even explored as a potential biomarker for potential risk transmission, not only in the context of neuropsychiatric, but also metabolic disease [12, 49]. Our results show that injection of sperm RNA from Dex exposed fathers induces alterations in glucose tolerance, weight, hormonal stress response and behavior, but no alterations in insulin tolerance. Importantly, this phenotype differs from the phenotype observed in the offspring generated via IVF with sperm from Dex exposed males, that showed opposite BMI alterations, and a change in insulin tolerance. We thus urge cautiousness when proposing altered sperm RNA as a predictor of phenotypic outcome, since depending on the model, other sperm epimodifications might be more deterministic or required to work in concert with a modified RNA payload. This interpretation corroborates finding from rodent models investigating endocrine disrupter exposure relevant for trans-generational effects on metabolic health [28].

Accordingly, we show that mimicking stress pharmacologically via Dex injection induced changes in sperm chromatin accessibility at several loci. One locus appeared to be of particular interest, due to its overlap with a gene with potential relevance to the previously described metabolic phenotype, observed in offspring generated using IVF with the sperm of Dex exposed males [5]. The metabolic alterations included changes in insulin resistance, glucose tolerance and BMI. The affected gene, *Sos2*, is known for its potential to regulate insulin signaling [34]. We observed significant gene expression changes when manipulating the region affected by accessibility changes in sperm and therefore concluded this region to be capable of regulating the gene’s expression. Consistently, we showed altered *Sos2* expression in the offspring. This change was independent of increased sperm RNA in Dex treated males since the expression change was not present in the offspring resulting from RNA injections. A decrease in specific sperm RNAs is not captured by total RNA injection, therefore we cannot exclude that a specific RNA with decreased abundancy in the sperm of Dex treated fathers contributes to the effect of *Sos2* expression change in the offspring. Yet in the absence of evidence of any putative role of any of the decreased miRNAs in the sperm of Dex exposed fathers, e.g. miRNAs known to regulate *Sos2*, we propose that the chromatin change in sperm primes an alternative trajectory that translates into altered gene expression at this very locus later in adulthood. The absence of *Sos2* gene expression changes in offspring resulting from RNA injections coincided with an absence of altered insulin signaling. While this might suggest a contribution of altered *Sos2* levels in the observed change in insulin signaling in IVF offspring, we by no means can exclude other important regulatory mechanisms of the complex process of insulin tolerance.

Our results further reveal that Dex decreases accessibility at 5fC sites containing the GRE motif in sperm, indicating a potential relay for glucocorticoid linked remodeling. GR can induce a conversion of condensed heterochromatin, indicative of transcriptionally silent regions, to more relaxed euchromatin, thereby facilitating gene expression at numerous promoters in somatic cells [50, 51]. Chromatin relaxation is not only important for the induction of gene expression but also a prerequisite for histone-protamine exchange [52]. It depends on histone H4 hyperacetylation via recruitment of histone acetyl transferases (HATs). Recruitment of HATs can be mediated by GR dependent and/or prior to chromatin remodeling [53]. Our finding of decreased activity at the GRE-related motif archetype supports the idea of GR dependent remodeling in sperm. Less GRE activity would indicate less histone-protamine replacement and hence result in altered histone retention and thus accessibility. This finding adds to the multiple factors described to be implicated in histone to protamine transition [54–61]. Interestingly, direct determinants of the small proportion of retained histones and associated accessible chromatin sites remain largely elusive. One exception concerns the involvement of the transcription factor CTCF. It is enriched in enhancers and promoters in sperm, which are also hotspots for histone retention. Studies using a conditional CTCF knockdown before male germ cells become haploid show failure in histone H2B retention [62]. However, not all histone retention sites contain CTCF pointing towards other important determinants of retention.

Our study explores 5fC as a potential contribution factor in defining histone retention and associated chromatin accessibility in sperm at functionally relevant TSS. 5fC was previously thought to merely mark sites that undergo active demethylation [63]. However, Raiber et al. showed that 5fC confers specific structural (flexibility) features to the DNA double helix suggestive of additional functions including chromatin remodeling [63]. Interestingly, 5fC can form covalent bonds with histones [64, 65]. Increasing 5fC by knocking out the gene responsible for base-excision repair to unmethylated cytosine in mouse ESCs reveals that histone lysines form Schiff base pairs at some 5fC sites in genomic contexts [64, 66]. This base pairing is supposedly present in mature sperm since native ChIP of histone modifications consistently shows histone retention in sperm [67] despite the absence of crosslinking otherwise required to retain histones prior to sonication and during pull down. 5fC immunohistochemical signal decreases with increasing maturity of sperm cells in the testis [68] as they slowly undergo histone replacement. Concomitantly, 5fC levels as assessed by mass spectrometry are particularly low in sperm coinciding with low histone levels in comparison to other cell types [36, 69] and with other 5-Cytosine modifications such as 5-Methylcytosine [40] and 5hmC [40, 41, 70]. Zhu et al. observed a proportion of “inherited” 5fC sites from sperm to the embryo reminiscent of the small proportion of inherited paternal histones [36].

Our results on higher accessibility at TSS at 5fC sites in comparison to TSS lacking 5fC partially support the idea of 5fC playing a role in chromatin remodeling in sperm. Yet upon lowering TDG levels using a heterozygous knockout and thereby putatively increasing the loci that retain 5fC we observe primarily decreased accessibility, defying the idea that, at these loci, 5fC itself instructs the retention of histones in the densely packed sperm chromatin in a linear fashion. It seems more plausible that 5fC sites can act as relays for TDG that usually can recruit remodelers and when no longer present will lead to a redirection of remodelers to distinct sites, thereby causing both increase and decrease in accessibility.

Generally, it has been reported that open chromatin regions in sperm are enriched in promoters hinting towards a potential priming of early embryonic transcription. Additionally, sperm 5fC regions shared with oocytes are depleted of promoters, and also sperm-specific 5fC regions are enriched at promoter regions [35]. Accordingly, 5fC in nucleosome-free regions in the early embryo have previously been shown to prime active demethylation in preparation for transcription [71]. We thus speculate that 5fC sites in sperm could influence early embryonic transcription, which seems to occur following an increase of 5fC in both the male and female pronucleus synergistically [36]. Whether environmental impact can affect 5fC levels themselves and thus early embryonic transcription remains to be determined.

In summary, our findings demonstrate the malleability of chromatin accessibility in sperm by extrinsic environmental (Dex treatment) and intrinsic (*Tdg* ± ) factors involving glucocorticoid signaling. We demonstrate an association between a paternal Dex induced change in gene expression of *Sos2* and altered chromatin accessibility in parental sperm suggesting a novel epigenetic mechanism of intergenerational transmission. Sperm RNA injection experiments induce phenotypic alterations in the resulting offspring that do not recapitulate the phenotype observed in animals generated using IVF with sperm from Dex exposed fathers. We conclude that the transmission of an aberrant phenotype in this model does not solely rely on altered sperm RNA, and that this likely can be extrapolated to the complex mechanism of inheritance of other environmentally induced phenotypes. Importantly, we introduce sperm chromatin accessibility as a relevant factor for stress induced intergenerational neuropsychiatric disease risk.

## Methods

### Animals

C57BL/6 J age and weight matched mice were born at the ETH Phenomics Center from the in-house breeding colony and acclimatized to our holding room for one week prior to the start of experiments. Animals were kept in single ventilated cages in groups of 4–5 on a 12 h reversed light dark cycle throughout experiments. Offspring from RNA injections were transferred to our animal facility after weaning. Weaning took place after postnatal day 21 and exact dates and litter sizes were recorded. Animals received standard chow (Granovit, Kaiseraugst, Switzerland, 3437) after weaning. RNA injection offspring were fed breeding chow (Granovit, 3802) until weaning. All tests were approved and performed under Cantonal commission license ZH222/19. *Tdg* ± animals used for sperm chromatin analysis were MGI:5487834 and for mouse embryonic stem cell generation MGI:7450955. Sample size was chosen based on previous studies.

### Dexamethasone paradigm and sample collection

Eight sexually mature male C57BL/6 J mice (12 weeks old) were injected with 2 mg/kg Dexamethasone (Sigma, St.Louis, Missouri, United States, D4902) dissolved in 10% DMSO (Sigma, 472301) and 0.9% saline (Moltox, Bone, North Caroline, United States, 51-40S022.052) and eight control males were injected with vehicle consisting of 10% DMSO and 0.9% saline. Animals were sacrificed 14 days later for sperm collection. Cauda epididymis and vas deferens were minced and placed in 37 °C warm M2 medium (Sigma, M7167) for at least 15 min. Sperm cells were separated from contaminating tissue by filtration through a 70 um nylon mesh (Corning, Corning, New York, United States, 431751) using several rinses with 1× PBS (Gibco, Waltham, Massachusetts, United States, 10010-015). Cells were inspected for impurities under the microscope before cell counting and aliquoting.

### In vitro fertilization and offspring generation

In vitro fertilization was carried out at the research support facility at Sanger institute using their standard procedures as described previously [5] to produce a cohort of offspring derived from sperm of Dexamethasone or vehicle injected males.

#### RNA injections

The sperm RNA from each treatment group (*n* = 6 each) were pooled separately and injected at a concentration of 0.5 ng/l into the pronuclei of naïve fertilized oocytes of control females following superovulation. Surviving embryos were transferred into the oviduct of pseudopregnant Swiss-NMRI foster mothers and allowed to develop to term. The experimenter was blinded to the treatment. The RNA injection process had to be carried out in two batches (each consisting of both treatment groups), to ensure enough offspring were produced. This resulted in the formation of two age groups within the testing cohort, which were a month apart in age. The two RNA injection cohorts were weighed once a week for 5–6 weeks, exact age at time of measurement was recorded. For all metabolic and behavioral tests, 16 male and 16 female offspring were tested, and half of each sex group were RNA injection offspring and the other half vehicle injection offspring, resulting in a number of 8 animals per group and sex.

### Metabolic tests

Animals were single housed in the morning of each testing day and cage food was removed in order to fast subjects for five hours before testing began at 02:00 pm.

#### Glucose tolerance test and insulin tolerance test

Mice were intraperitoneally injected with a 0.3 g/ml glucose solution (Sigma, 7528) in 0.45% saline to achieve 2 mg/g body weight for glucose tolerance and 1 mU insulin/g body weight (Novo Nordisk, Bagsværd, Denmark, 00536752) for the insulin tolerance test. They were then placed under a large glass beaker, and their tails were taped to the bench and their tail was punctured. Blood glucose concentration was measured using a blood glucose meter (Accuckeck aviva, Roche, Basel, Switzerland). Measurements were taken at 0-min, 15-min, 30-min, 90-min and 120-min timepoints. After the 30-min time point, each subject was returned to their single housing cage where the remaining measurements were taken.

#### Corticosterone response to stress

Animals were restrained for 30 min in 50 ml plastic centrifuge tubes with a hole in the tip and cap for air circulation and tail protrusion. Blood sampling was performed from tail prick at 0-min, 15-min, 30-min, 90-min and 120-min timepoints. After overnight storage at 4 °C to allow for natural clotting, the samples were centrifuged at 2500 × *g* for 10 min at 4 °C. The serum supernatant was collected, pipetted into fresh Eppendorf tubes and frozen at −80 °C.

### Behavioral tests

Subjects were singled housed the day before testing. All behavioral tests were video recorded and carried out in TSE Multi Conditioning Systems for 10 min, after which animals were returned to their single housing cages before regrouping. Before each test, the subject was placed into the test area, the floor and walls of the test apparatus was cleaned with 1 nM soap solution. The Open Field Test took place under 4 lux light intensity in an arena of 45 cm length, 45 cm width and 40 cm height. The Light Dark Box Test was carried out in an arena consisting of a light compartment with dimensions 28 cm length, 30 cm width, 25 cm height and a dark compartment with dimensions 16 cm length, 30 cm width, 25 cm height. Test subjects were able to cross between these two compartments via an opening in the divider between the compartments. Mice were placed into the light compartment at the start of the test. Videos were then analyzed using animal pose estimation software DeepLabCut [72].

### Necropsy

The IVF derived animals used for necropsy of liver samples had undergone phenotyping as described previously [5]. Necropsy for RNA injected offspring was carried out on 16 males and 16 females, which had undergone all experiments outlined above. Liver samples were dissected and snap-frozen in liquid nitrogen before transfer for storage at −80 °C until processing.

### Corticosterone ELISA

Corticosterone in the serum from blood samples taken during the glucose response to restraint experiment was measured using an ELISA kit (Assaypro, St. Charles, Missouri, United States, EC3001-1). 2 ul of each serum sample was diluted by a factor of 100 in SBS diluent and subsequently the ELISA was carried out in duplicates and according to the manufacturer’s instructions.

### Cell culture and transfection

Neuro-2a (N2a) neuroblastoma cells were cultured in DMEM (Gibco, 41965039) containing glutamine and glucose with 10% fetal bovine serum and 1% penicillin-streptomycin (Gibco, 15140122) and passaged every 3 days. For experiments, 100,000 cells per well were seeded in 12-well plates. The following day, 85% confluent cells were washed with 1× PBS and transfected with 1 ug of a plasmid encoding dCas9-KRAB fusion protein (pLV hU6-sgRNA hUbC-dCas9-KRAB-T2a-Puro, Addgene, Watertown, Massachusetts, United States, 71236). Transfection was performed using lipofectamine2000 (Invitrogen, Waltham, Massachusetts, United States, 11668019) in serum-free culture medium and overnight. The following day, cells were washed with 1× PBS and cells selected in culture medium with 1 ug/mL puromycin (Sigma, P8833) for 2 days. Cells were washed with 1× PBS, then transfected with a 1:1 preincubated mix of 100 ng crRNA (5-AAGGAGTGTATACTGTGTAA-3′) and 300 ng tracrRNA (Microsynth, Balgach, Switzerland) in serum-free medium using lipofectamine2000 overnight. The next day, cells were washed with 1x PBS and harvested with 1 mL TryplE (Gibco, 12604013), then snap-frozen in liquid nitrogen.

### RNA extraction and q-RT-PCR

RNA was extracted from liver and kidney samples using TRIzol (Invitrogen, 15596-026) according to the manufacturer’s protocol after homogenizing them in a tissue lyser bead mill (Qiagen, Venlo, The Netherlands) at 4 °C for 2 min. The RNA samples were removed of any contaminating DNA by carrying out a DNase treatment, using the Ambion DNA-*free* Kit (Invitrogen, AM1906), according to the manufacturer’s instructions.

RNA of snap-frozen N2a cells were extracted with the RNeasy Plus Mini Kit (Qiagen, 74134), which includes a column-based gDNA elimination step, according to the manufacturer’s protocol. The concentrations of the RNA samples were quantified using a UV/V spectrophotometer (Nanodrop 1000, Thermo Fisher Scientific, Waltham, Massachusetts, United States).

900 ng of each RNA tissue sample and 1000 ng of each RNA cell sample were converted to cDNA using M-MLV Reverse Transcriptase (Promega, Walldorf, Germany, M1705) and Oligo(dt) 15 primers (Promega, C1101) containing RNasin® Plus RNase inhibitor (Promega, N2615) according to the manufacturer’s recommendations. The resulting cDNA samples were diluted 1:4 and 2 ul used for q-RT-PCR. Signal was amplified using SYBR Green (Roche, 4887352001) using 90 °C for 5 min, 51 cycles of 95 °C for 10 s, 60 °C for 10 s, 72 °C for 10 s and plate reading, followed by 95 °C for 5 s, melt curve at 65 °C to 95 °C in 0.5 °C increments for 5 s and 40 °C for 30 s, reading the signal out using a thermal cycler (Bio-Rad, Hercules, California, United States). *Sos2* forward primer 5-ACC ATC TTT GCT CCA GTC CTC TT-3′ and reverse primer 5′- GGT GGA ATA GCA GGA GGG TCA-3′. Endogenous control *Tubd1* forward primer 5′-TCT CTT GCT AAC TTG GTG GTC CTC-3′ and reverse primer 5′-GCT GGG TCT TTA AAT CCC TCT ACG-3’ (all Microsynth).

### ATAC library preparation

For Dex vs. Veh 100 000 fresh 1× PBS washed sperm cells were incubated on ice for 3 min in resuspension buffer (0.1% v/v NP-40, 0.1% v/v Tween-2 and 0.01% v/v Digitonin, 10 mM Tris-HCL pH7.5, 10 mM NaCl, 3 mM MgCl2), after a washing step (10 mM Tris-HCL pH7.5, 10 mM NaCl, 3 mM MgCl2) nuclei were recovered by centrifugation (500 × *g*, 10 min, 4 °C). Nuclei were transposed in tagmentation buffer supplemented with Tween-20 0.1% v/v, Digitonin 0.01% v/v and 2.5 ul Tn5-Transposase for 30 min at 37°C while shaking (1.000 rpm). DNA was purified using the MinElute Reaction Cleanup (Qiagen, 28204) and amplified using the NEBNext high-Fidelity 2X master mix (New England Biolabs, Ipswich, Massachusetts, United States, M0541) and 2.5 uM Nextera primers (Illumina, San Diego, California, United States, FC-141-1007) at 72 °C for 5 min, 98 °C for 30 s and 6 cycles of 98 °C for 10 s and 63 °C for 30 s, followed by 1 min at 72 °C. A subfraction of the amplification product was then subjected to q-PCR to evaluate how many additional PCR cycles would be required. Remaining PCR product was then amplified for additional cycles before double sided bead purification with AMPure XP beads (Beckman Coulter, Brea, California, United States, A63880). Libraries were inspected quantitatively and qualitatively on an Agilent 2100 Bioanalyzer (Agilent Technologies, Santa Clara, California, United States). Sequencing aimed at 50 Million reads/sample. The same protocol was used for *Tdg* ± and wildtype control sperm, but instead of Nextera primers, Diagenode primers (Diagenode, Marlborough, Massachusetts, United States, C01011034) were used, and sequencing was aimed at 100 Million reads/sample.

### Mouse embryonic stem cell (ESC) generation, culture and RNA sequencing

Embryonic stem cell lines were rederived and cultured in serum-free medium as described previously [39]. 10 male ESC lines were obtained from 5 simultaneously timed matings of Tdg ± male and female. Blastocysts were cultured on mitotically inactivated murine feeder cells for 3–4 passages in DMEM supplemented with 15% KO serum replacement (Gibco, 10828028) and leukemia inhibitory factor LIF (Merck-Millipore, Darmstadt, Germany, LIF1010) before adaptation to 2i medium [73]. Total RNA was extracted from rederived ESC for approximately 10–12 passages after removal of feeder cells for three passages. Total RNA libraries were prepared using the Illumina® Stranded Total RNA Prep with depletion of ribosomal RNA (Illumina, 20040529) according to manufacturer’s recommendations. RNA sequencing (SE80) was carried out on a NextSeq 500 platform.

### Data analysis

#### Phenotyping

SPSS was used to compute statistics of metabolic and behavioral tests, as well as corticosterone tests. We tested for the contribution of several covariates to the overall variance of the data. These included: litter size [74], day of testing, age [75], and age at weaning. If the covariate was statistically significant (*p* < 0.5) in at least one sex and the eta squared exceeded 5%, then the covariate was added to the statistical model to improve its accuracy. Uncorrected data are also made available and respective plots available under supplementary material. We did not exclude any data points. When appropriate (GTT, ITT, Glucose in response to restraint, Corticosterone in response to restraint) repeated measurement ANOVA was applied. Behavioral data were analyzed with two-way ANOVAs, using sex and treatment as independent variables. Significance level was set to *p* < 0.05.

Gene expression data generated via q-RT-PCR of IVF offspring and dCAS-KRAB experiment were analyzed using Mann-Whitney nonparametric test in GraphPad Prism, since data were not normally distributed. Data were assessed for homogeneity. Data from RNA injection offspring were normally distributed and variances did not differ between groups hence were analyzed with a two-tailed Student’s *t*-Test.

#### ATAC sequencing analysis

Adapters were trimmed with trimmomatic, and reads were aligned to the GRCm38.p5 genome using Bowtie2 (2.3.4.3) [76] end-to-end alignment (with --no-unal -X 2000 --no-mixed --no-discordant --score-min L,−0.4,−0.4). Duplicated fragments were marked with Picard (2.18.27) [77], chrM and ENCODE blacklisted regions were excluded, and fragments with MAPQ > = 30 and length between 50 and 115 (40 and 120 for wt and *Tdg* ± data) were retained as nucleosome-free reads. For Dex vs Veh treated samples the retained reads were shifted and merged across samples before running MACS 2.2.5 [78] (BAMPE mode) to identify nucleosome-free regions. One sample was excluded because it presented poor QC (high duplication rate, poor enrichment for TSS). Sample-level quantification of the regions was performed using featureCounts 1.6.4 (with -p --ignoreDup --primary) [79] on all fragments (irrespective of size). Differential accessibility analysis on the region was performed using edgeR (3.27.13) [80] with the exact test, testing regions with more than 10 reads in more than 1 sample. For *Tdg*± vs. wildtype MACS (BAMPE mode) was used on all fragments to identify broadPeaks per samples, which we defined as “accessible” regions. NarrowPeaks per sample were generated after shift and removal of the blacklisted regions and defined as “nucleosome-free accessible” (NFA) regions per sample. Total consensus “accessible” regions were defined as being present in at least 2 of all 5 samples. *Tdg* ± consensus “accessible” regions and “NFA” regions were defined as being present in at least 2 of the 3 *Tdg* ± replicates.

#### Motif analysis

Motifs were obtained from the HOCOMOCO v11 core motif database, and genome-wide hits for motif archetypes were downloaded from a previously published study [81]. Motif enrichment analysis on sperm total 5fC sites or 5fC sites in proximity to a TSS (±2kB) was performed with memes 0.99 and MEME Suite (- size 200) [82], with different sets of control regions. For motif enrichment analysis on the archetype level a two-sided hypergeometric test was used to test over-representation of motif archetypes in sets of regions. For differential motif activity analysis, per-sample motif-wise scores were obtained from ATAC-seq data using chromVAR 1.12 (size -250 for NFA, 200 for 5fC/no5fC and TSS) [83], and the resulting scores were z-score normalized before running differential analysis with limma 3.46 [84].

#### RNA-seq analysis

RNA-seq data was quantified with Salmon v0.8.1 [85] on the GRCm39 v108 transcriptome.

Surrogate Variable Analysis was used on variance-stabilized expression data using the sva package SEtools (1.9.3) [86] with 3 surrogate variables. The corrected expression data of genes with at least 20 reads in at least one sample were used to inferred TF activity using the viper 1.24 package [87] with regulons from DoRothEA [88], followed by differential analysis using limma [84].

Methylated sperm promoter regions were extracted from publicly available data GSE75323. The 5fC data of mouse sperm (GSE84833) was lifted to mm10 via liftOver [89]. For no5fC sites the same sequencing data was used by extracting sites where no 5fC was measured and are not in proximity to 5fC sites (±2kb) as well as unique (±1kb to other no5fC sites). TSS with a 5fC site ±2kb were declared as TSS with 5fC. TSS annotation GRCm38 Ensembl v102.

Signal heatmaps were generated using epiwraps 0.99 [90] with background normalization. Signal clusters were generated with k-means clustering.

### Supplementary information

Supplementary Figures

## Data Availability

Sequencing data generated in this manuscript have been submitted to the GEO repository under the following accession numbers: GSE233892 and GSE234039. Code has been deposited on the Github repository: [https://github.com/ETHZ-INS/Fischer-Kretschmer-et-al.-2023]. All remaining data have been archived in ETHs research data collection under accession number: 10.3929/ethz-b-000615271.
