# Supplementary material for: Sperm chromatin accessibility’s involvement in the intergenerational effects of stress hormone receptor activation
Source: Transl Psychiatry. 2023 Dec 8;13:378. doi: 10.1038/s41398-023-02684-z (PMC10709351; doi:10.1038/s41398-023-02684-z)
Supplement: Supplementary file 1 — Supplementary Figures [file 41398_2023_2684_MOESM1_ESM.docx]

**Sperm chromatin accessibility's involvement in the intergenerational effects of stress hormone receptor activation**

**Supplementary information**

Vincent Fischer^1,2*^, Miriam Kretschmer^1,2*^, Pierre-Luc Germain^1,3,4,5^, Jasmine Kaur^1^, Sergio Mompart-Barrenechea^1^, Pawel Pelczar^6^, David Schürmann^7^, Primo Schär^7^, Katharina Gapp†^1,2^

^1^ Laboratory of Epigenetics and Neuroendocrinology, Institute for Neuroscience, Department of Health Sciences and Technology, ETH Zürich, Switzerland

^2^ Neuroscience Center Zurich, ETH Zurich and University of Zurich, Switzerland

^3^ Laboratory of Behavior and Molecular Neuroscience, Institute for Neuroscience, Department of health science and technology,

^4^ Computational Neurogenomics, Institute for Neuroscience, Department of health science and technology,

^5^ Lab of Statistical Bioinformatics, University of Zürich, Switzerland

^6^ Center for Transgenic Models, University of Basel, Basel, Switzerland

^7^ Department of Biomedicine, University of Basel, Mattenstrasse 28, Basel, 4058, Switzerland

* These authors contributed equally.

† corresponding author: katharina.gapp@hest.ethz.ch


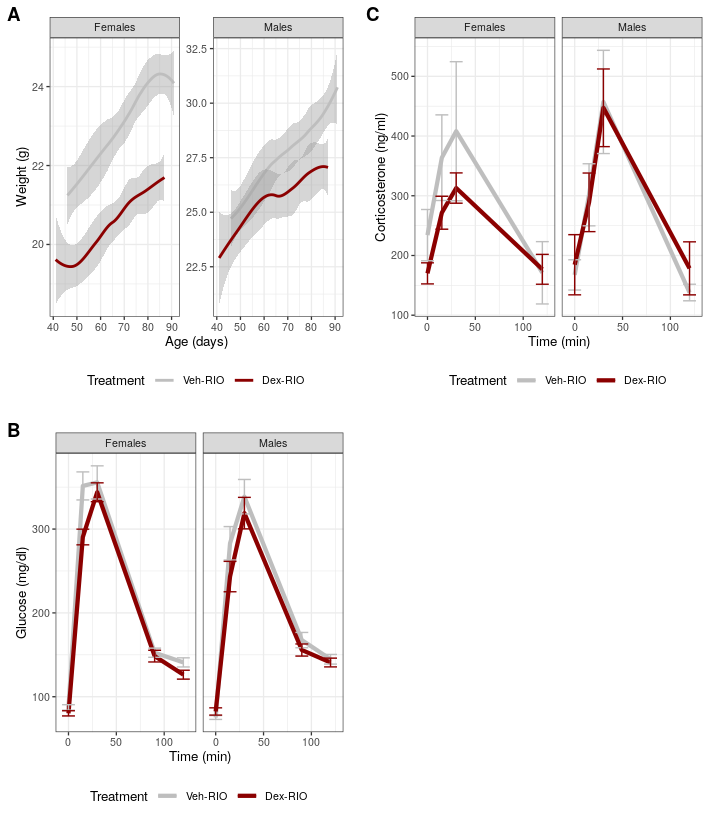


**Supplementary Figure 1.** Phenotyping results of RNA injection offspring (RIO) without considering covariates of A) body weight, B) glucose in response to an injection of glucose. Using the day of testing as a covariate, repeated measures ANOVA showed a significant effect of treatment (F(1, 58)=5.548, p=0.022) on the response to a glucose injection in Dex-RIO versus Veh-RIO. We also observed a time-treatment interaction (F(2.194, 127.229)=3.952, p=0.018) and a time-sex interaction (F(2.194, 127.229)=8.576, p=0.000) C) Corticosterone in response to and during the recovery of restraint (statistical details see main text).


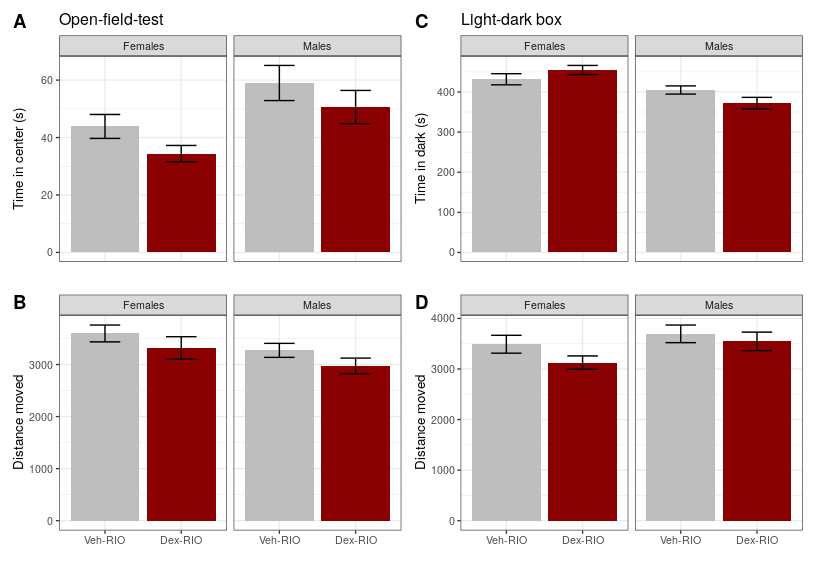


**Supplementary Figure 2.** Behavioral phenotyping results of RNA injection offspring (RIO) depicted without considering any covariates. A) We observed a significant effect of treatment on time spent in the center using age cohort as a covariate (F(1, 58)=7.322, p=0.009) in the open field test. Dex-RIO animals showed a reduced time spent in center compared to Veh-RIO, indicating an anxiogenic effect. Furthermore, we observed a significant effect of sex (F(1, 58)=13.161, p=0.001), with females spending less time in center than males, suggesting higher anxiety levels in females. B) We also found a significant effect of treatment on distance covered in the open field test using age cohort as a covariate (F(1, 58)=4.904, p=0.031). C) When analysing the time spent in the dark compartment of the light dark box test with no significant covariates, we found a significant sex-treatment interaction (F(1, 59)=4.957, p=0.030). When splitting the analysis by sex we further observed that Dex-RIO males showed a strong trend towards spending less time in the dark compartment than controls (F(1, 29)=3.491, p=0.072), while in females we did not observe such effect (F(1, 30)=1.657, p=0.208) and D) In concurrence with a potential overall reduction in activity, two-way ANOVAs showed that Dex-RIO animals exhibited a lower distance moved (F(1, 57)=6.182, p=0.016) than Veh-RIO using age-cohort and age at weaning as covariates in the light dark box test.

A B


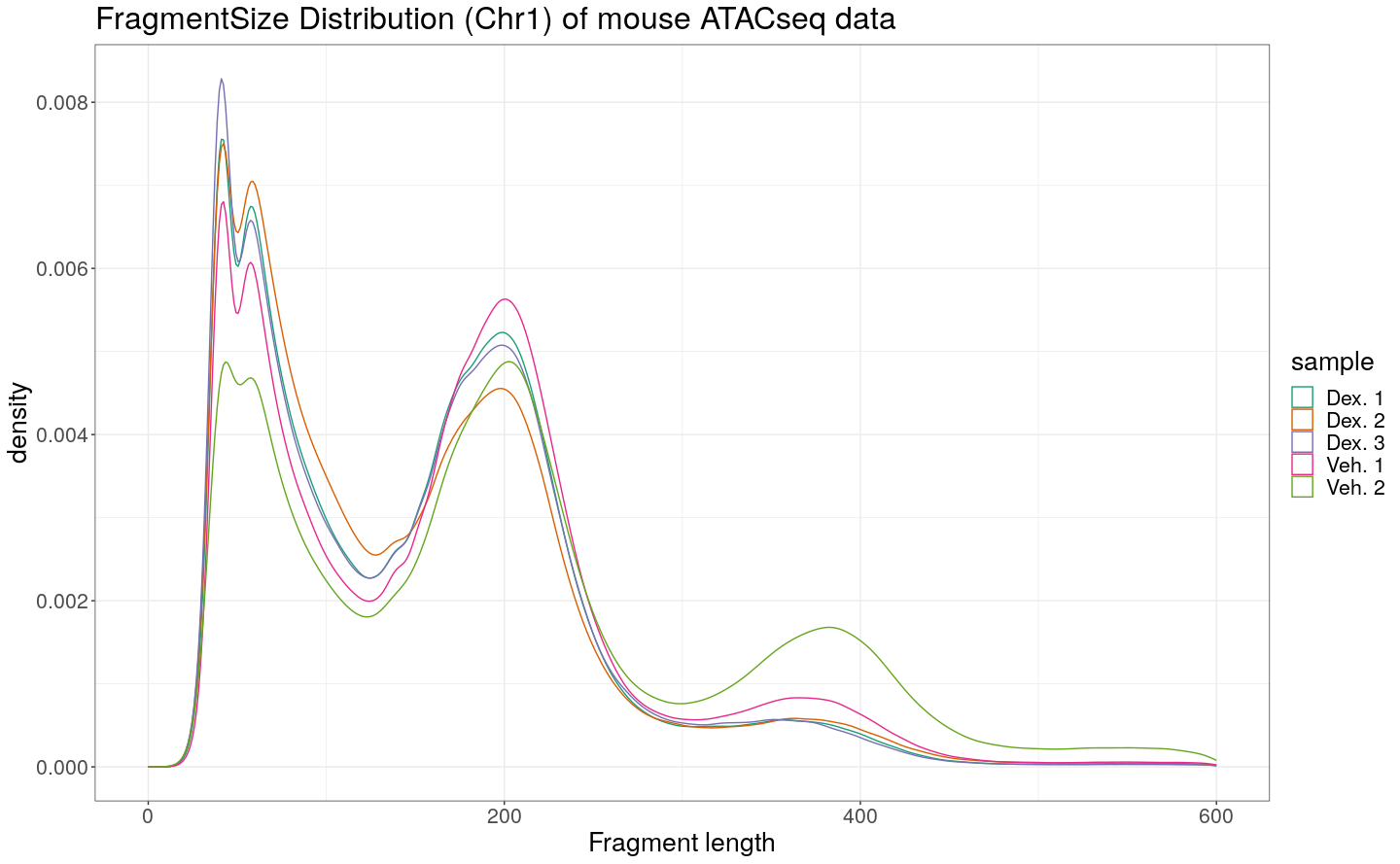

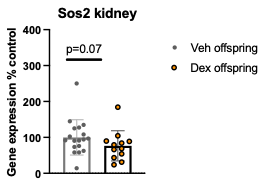


**Supplementary Figure 3.** A) Distribution of ATAC-seq fragments from animals with Veh or Dex treated sperm.

B) *Sos2* expression in kidney of offspring of males injected with Veh or Dex (U=65).


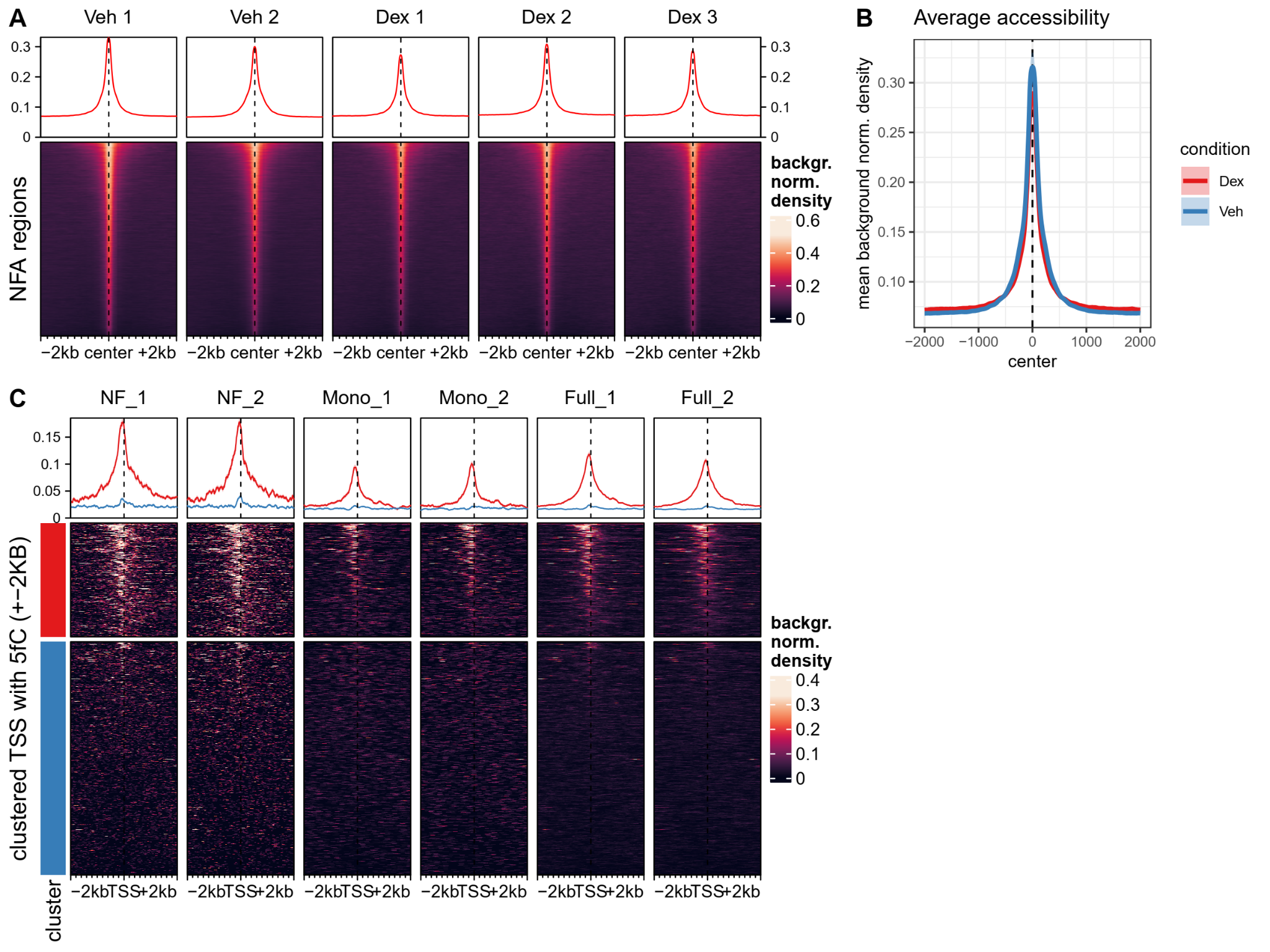


**Supplementary Figure 4.** ATAC-seq data from sperm of age matched males 2 weeks post injection of Dex or Veh. (A) Individual heatmaps of each sample (B) Average accessibility of nucleosome-free regions Dex vs Veh (C) Heatmap depicting a clustering of ATAC peaks based TSS with 5fC sites in proximity. NFA regions= nucleosome-free accessible regions.


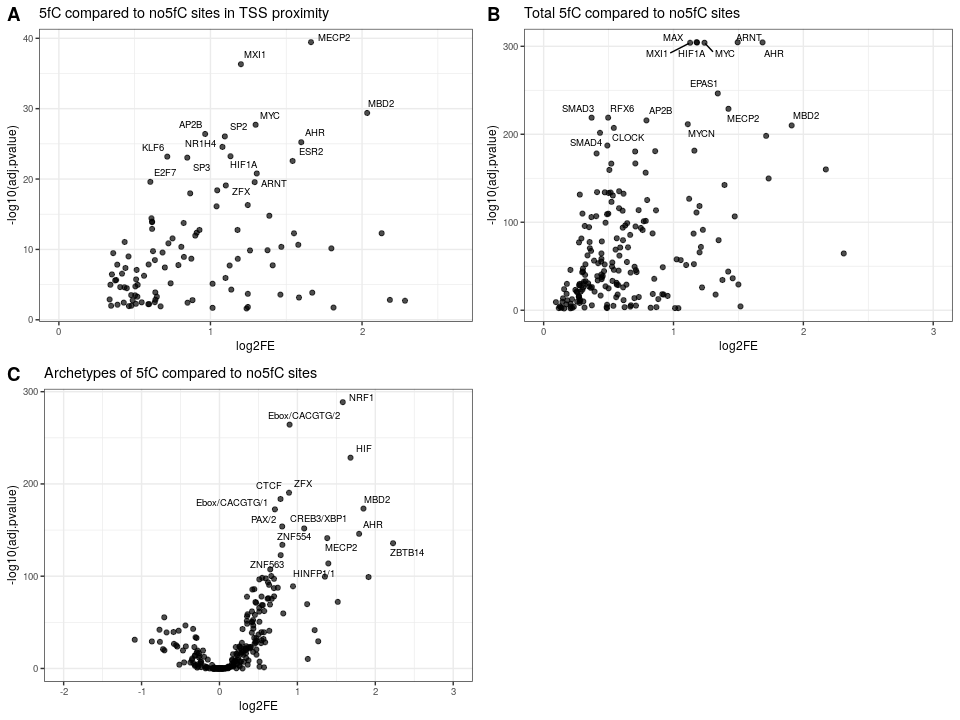


**Supplementary Figure 5.** Transcription factor motif enrichment analysis of (A) 5fC sites in proximity to a TSS versus no 5fC sites in proximity to TSS mouse sperm, (B) of all 5fC sites in mouse sperm, (C) of all 5fC sites in mouse sperm at motif archetype level. Top 15 significant motifs are displayed.


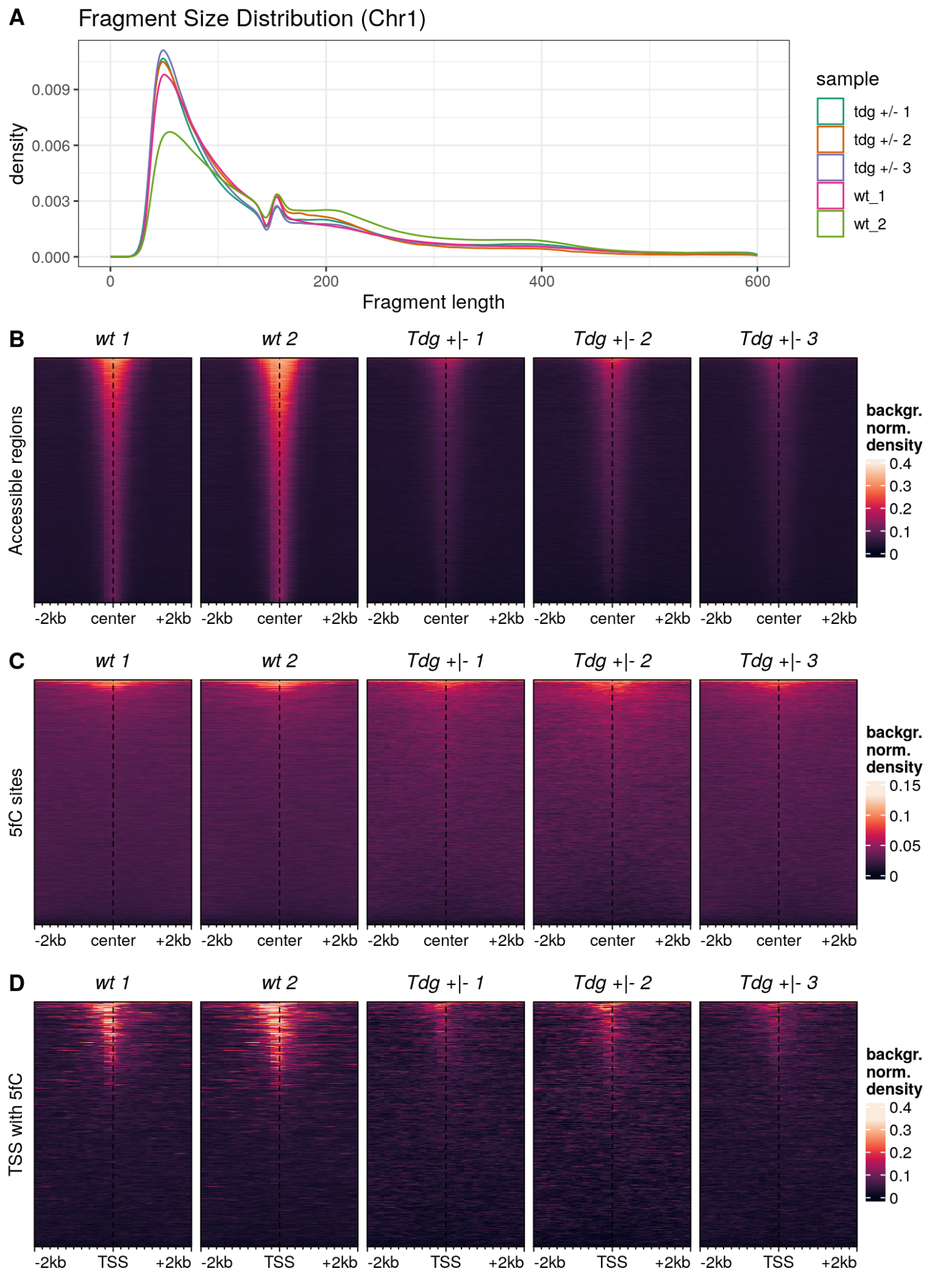


**Supplementary Figure 6.** (A) ATAC-seq data size distribution of mouse sperm of *Tdg*+/- males. (B) Accessibility changes in accessible regions in mouse sperm of *Tdg*+/- males based on consensus peaks (present in at least 2 replicates of either control or *Tdg*+/- samples). (C) Accessibility at 5fC sites in mouse sperm of *Tdg*+/- males (D) Accessibility change at TSS with 5fC in proximity in mouse sperm of *Tdg*+/- males on consensus peaks (present in at least 2 replicates of either control or *Tdg*+/- samples).


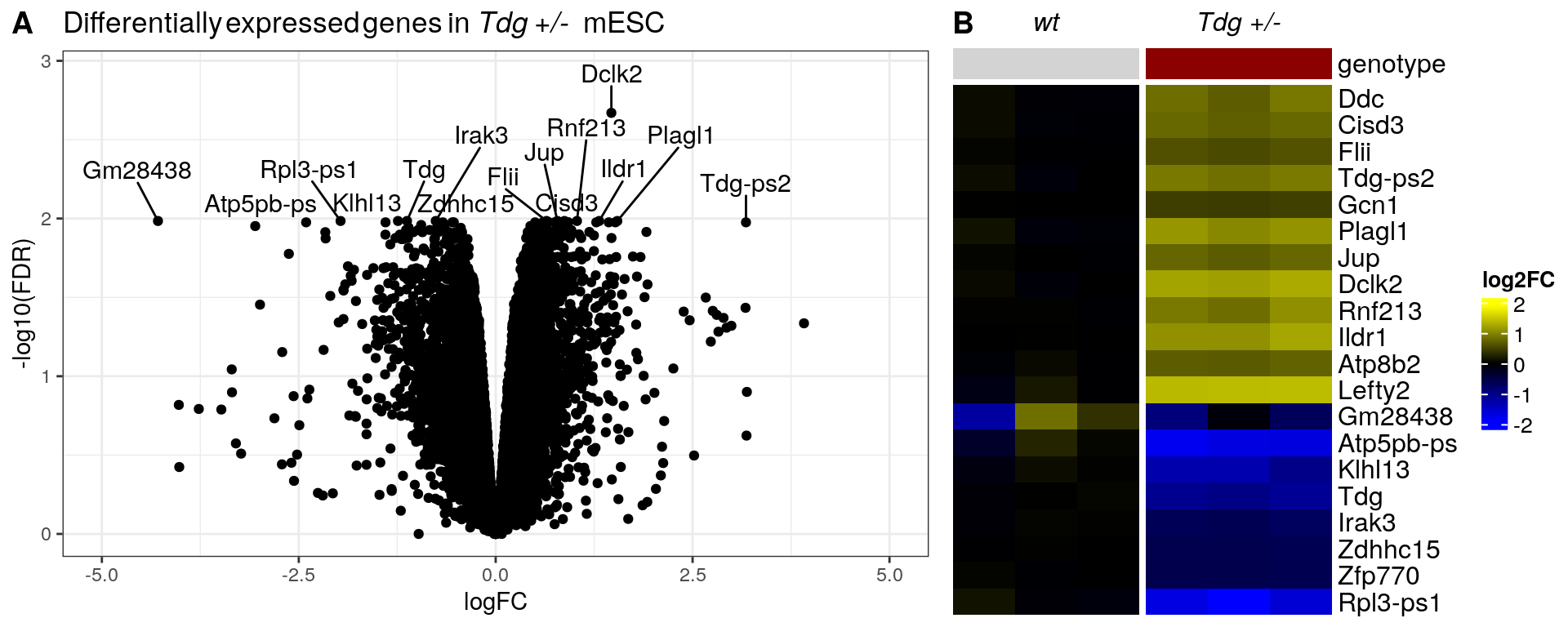


**Supplementary Figure 7.** RNA sequencing results from mouse embryonic stem cells of *Tdg+/-* versus control cells (n=3). A) Volcano plot and B) heatmap depicting differentially expressed genes. Data was adjusted with surrogate variable analysis. Top 15 (A) and Top 20 (B) significant genes are displayed.
